# Aging, Osteo-Sarcopenia, and Musculoskeletal Mechano-Transduction

**DOI:** 10.3389/fresc.2021.782848

**Published:** 2021-12-06

**Authors:** Jenna M. Leser, Anicca Harriot, Heather V. Buck, Christopher W. Ward, Joseph P. Stains

**Affiliations:** Department of Orthopaedics, University of Maryland School of Medicine, Baltimore, MD, United States

**Keywords:** bone, muscle, microtubules, mechanotransduction, sarcopenia, cytoskeleton, osteopenia, osteoporosis

## Abstract

The decline in the mass and function of bone and muscle is an inevitable consequence of healthy aging with early onset and accelerated decline in those with chronic disease. Termed osteo-sarcopenia, this condition predisposes the decreased activity, falls, low-energy fractures, and increased risk of co-morbid disease that leads to musculoskeletal frailty. The biology of osteo-sarcopenia is most understood in the context of systemic neuro-endocrine and immune/inflammatory alterations that drive inflammation, oxidative stress, reduced autophagy, and cellular senescence in the bone and muscle. Here we integrate these concepts to our growing understanding of how bone and muscle senses, responds and adapts to mechanical load. We propose that age-related alterations in cytoskeletal mechanics alter load-sensing and mechano-transduction in bone osteocytes and muscle fibers which underscores osteo-sarcopenia. Lastly, we examine the evidence for exercise as an effective countermeasure to osteo-sarcopenia.

## Introduction

With advancing age, a threshold of decline in the mass and function of bone and muscle marks the onset of musculoskeletal frailty. This loss of muscle and bone quality, collectively called osteo-sarcopenia, initiate changes in lifestyle, and activity levels that start a sequence of events that lead to significant morbidity and even mortality. Indeed, muscle and bone health are remarkably predictive of biological health. Muscle mass or performance, including grip strength and gait speed, and bone mass or hip fracture have all been correlated to increased morbidity and mortality (1–10). Likewise, the incidence of low impact fractures steeply inclines in the osteo-sarcopenia population, contributing to reduced mobility and independence, increased mortality, and corresponding to increased financial burdens (10–13).

Both men and women experience peak bone mass around 30 years of age, with a steady decline for the rest of life thereafter (14). In parallel, muscle strength substantially declines with age followed by declines in muscle mass too (15). When overlaid, the loss of bone mineral density (BMD), muscle strength, and muscle mass, occurs concomitantly suggesting the processes happen in parallel, or are interdependent (16). Since bone and muscle are known to regularly remodel in response to ever changing mechanical cues, cellular dysfunction caused by aging results in a significant decrease in musculoskeletal formation, thus favoring catabolic processes leading to osteopenia and sarcopenia (osteo-sarcopenia).

**Figure 1:**
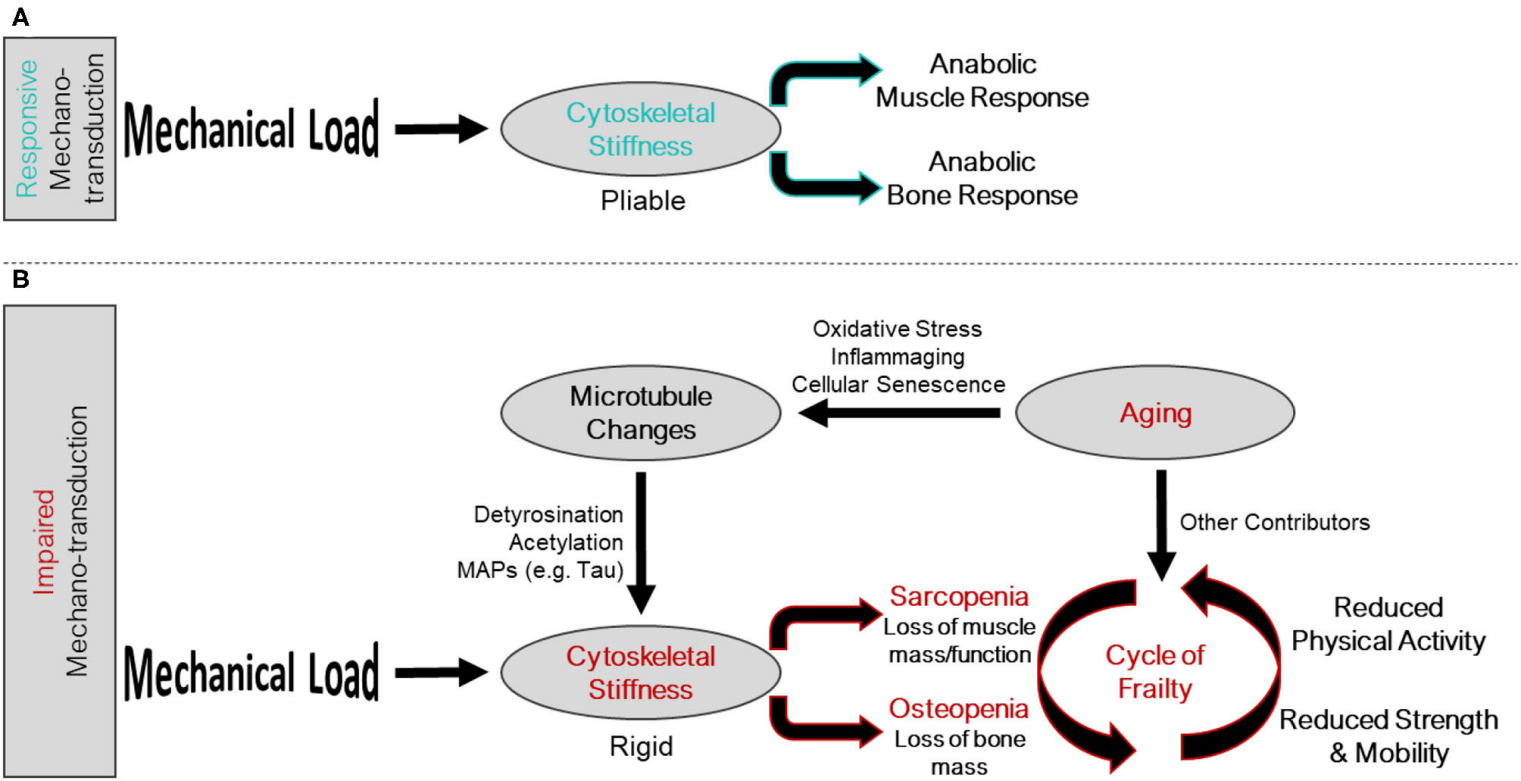
Intersection of the mechano-transduction pathway and the cycle of frailty. **(A)** Responsive mechano-transduction in youth. Mechanical load is sensed by a pliable cytoskeleton initiating the anabolic responses of both muscle and bone. **(B)** Impaired mechano-transduction in aging. Mechanical load is sensed by a rigid cytoskeleton, influenced by changes to the microtubule network, a consequence of aging related phenomena, leading to sarcopenia and osteopenia. These musculoskeletal pathologies feed into the cycle of frailty, where reduced strength and mobility, and reduced physical activity propagate the feed forward cycle.

Aging related changes to musculoskeletal tissues are multifactorial and include reduced activity levels, chronic low-grade inflammation, oxidative stress, and impaired autophagy, among others. Together, these factors conspire to initiate a feed forward loop of global changes in bone and muscle cell gene expression (17, 18), tissue composition, and tissue functions that exacerbates osteo-sarcopenia (19–21) termed the cycle of frailty (Figure 1). The initiation of the cycle of frailty poses a “chicken or the egg” paradox when it comes to reductions in activity, bone mass, and muscle mass. It has yet to be determined whether the onset of the cycle of frailty starts as aging-related changes to bone and muscle which cause individuals to become less active, or if inactivity causes aging-related declines in muscle and bone. What can be said with certainty is that the cycle is a feed-forward loop where inactivity, regardless of the reason, causes disuse of bone and muscle, thusly reducing strength, weakening balance, and increasing fracture risk, ultimately furthering inactivity. Accordingly, therapeutic targets to improve bone and muscle mass and function have tremendous potential to disrupt this feed forward cycle and lead to better patient outcomes.

**Figure 2:**
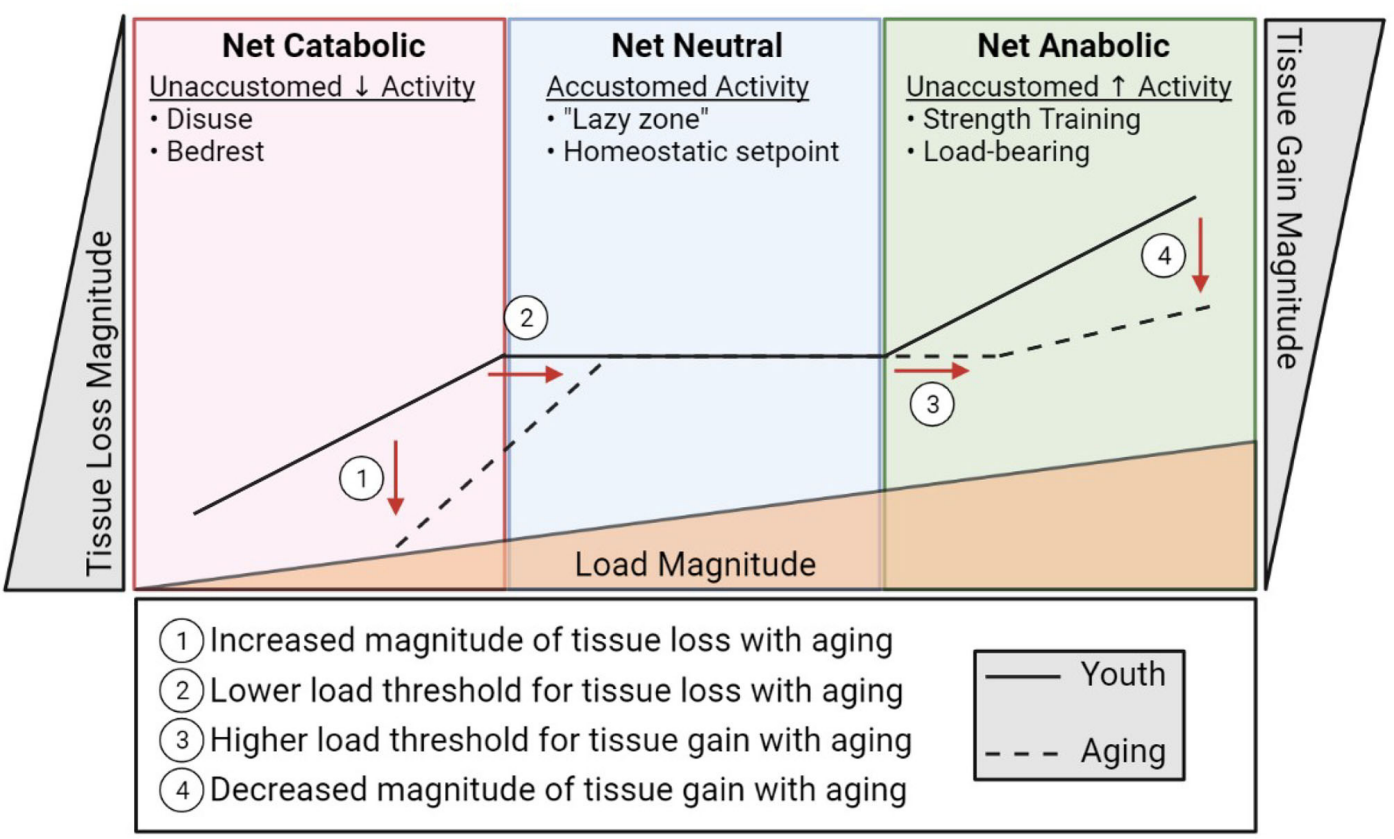
A diagrammatic representation of musculoskeletal catabolic, neutral, and anabolic zones. The solid line represents how load (bottom triangle) influences bone and muscle loss (left), maintenance (center), and creation (right) in youth. The dashed line indicates the apparent shift in load sensitivity (shifted right) and loss/gain magnitude (shifted up/down, respectively) associated with aging.

Bone and muscle sense and respond to their mechanical demands by adjusting their mass and quality. This permits the musculoskeletal system to adapt to changing activity levels to accommodate function. Muscle must be sufficiently robust to be able to mediate daily activities, which can vary dramatically based on lifestyle. Likewise, within this range of typical activity, bone must be sufficiently strong to bear load without failure (fracture). This ability of cells to sense mechanical events and translate them into biological signals that dictate cell and tissue function is termed mechano-transduction. At an accustomed amount of mechanical loading (i.e., typical daily activity and exercise) bone and muscle operate to maintain function around this homeostatic set point, maintaining and repairing the tissue. This homeostatic setpoint at which slight variance in activity does not prompt net increases or decreases in bone or muscle mass is sometimes referred to as the lazy zone (Figure 2). When muscle and bone experience unaccustomed loads such as a sudden, sustained change in lifestyle (e.g., a rigorous strength training regimen), the cells of these tissues sense and respond to this change activating a mechano-transduction pathway to enter an anabolic phase where new tissue is deposited to accommodate this new stimulus. Eventually, bone and muscle will fully adapt to the new mechanical demands and, if these demands remain sustained and static, this mechanical environment will become an accustomed load and a new homeostatic set point defined at this level of musculoskeletal mass and quality. The converse is true in disuse. A sustained decrease in activity and loading (e.g., bedrest) will be sensed by the cells of bone and muscle and initiate a catabolic phase where the musculoskeletal system adapts to its new mechanical environment by reducing bone and muscle mass, setting a new homeostatic set point around the adapted activity inactivity levels. Indeed, anyone with a limb immobilized in a cast for 12 weeks has experienced the rapid adaptation of muscle mass to disuse through atrophy.

An insidious feature of aging is that this exquisitely adapted ability to sense and adapt to a changing mechanical environment begins to unravel. In both bone and muscle, the ability to respond to loading is impaired with increasing age (22–24). In rodent models, the ability of bone cells to sense and respond to a mechanical load is reduced in old mice compared to sex matched young mice at various forces (24, 25). What this means is that a load capable of stimulating bone formation in young mice is not enough to stimulate bone formation in old mice, and in order to produce comparable new bone in old mice as in young mice, the load in old mice needs to be increased. Essentially, if we over simplify this to an example with weights on a squat bar, a young individual could build bone using ~200 lbs, whereas an old individual would have to use >300 lbs to build new bone. This is not feasible for a population of people who are also ailed by sarcopenia. Not only are osteocytes less stimulated by mechanical load in aging, they are also less capable of responding to the load that they are receiving.

Interestingly, initiation of sarcopenia associates aging with a decline in muscle strength prior to a decline in muscle mass, suggesting functional measures in muscle may be predictive of aging earlier than muscle size (15). Consistent with this concept is the evidence that functional measures of muscle (i.e., grip strength, gait speed) are strong predictors of biological age (15). Given that the hypertrophic response to resistance exercise is reduced in aging relative to youth, yet increases in strength and power are achievable (26–28), the mechano-biology of muscle mass is of primary concern.

Here, we will examine the provocative idea that aging related factors may disrupt the very machinery that bone or muscle cells use to sense and respond to mechanical stimuli. It is important to acknowledge that many of the mechano-sensors and mechano-transduction cascades in bone and muscle are affected by aging and are often interconnected at many levels (29–33). We will focus on an element of a recently described mechano-transduction pathway – the microtubule cytoskeleton – shared by bone and muscle cells, as an example of how aging related changes may influence mechano-transduction pathway (34–37). We think this is a reasonable model upon which to overlay these concepts in that nearly all the mechano-sensitive elements in muscle and bone have been linked directly or indirectly to the cytoskeleton and converge on calcium signals (38–40). Thus, many of these concepts can likely be extended to the other characterized mechano-sensors in musculoskeletal tissues. Additionally, we will survey the current literature suggesting how aging-related dysfunction of the cells in bone and muscle, and specifically their ability to sense and respond to mechanical cues, not only contribute to osteo-sarcopenia, but also may serve as mechanistic targets for future therapy. Finally, we will consider the impact of exercise as a mode of rehabilitation for the mechano-sensing apparatus in aged cells, restoring the homeostatic set point, and improving bone and muscle outcomes.

## Musculoskeletal Mechano-biology

The cells that facilitate mechano-responsiveness in bone and muscle are the osteocyte and the skeletal muscle fiber, or myofiber. Within these cells, numerous potential mechano-sensors, which have various levels of interconnection, have been identified (41–43), including focal adhesions, primary cilia, stretch activated ion channels, actin filaments, and microtubules (40). Mechano-sensors are the first step in sensing a change in accustomed load and translating mechanical cues into biological signals that regulate cellular function. While the multifactorial nature of aging undoubtably influences many of these mechano-sensors (and other aspects of cellular function) contributing to osteo-sarcopenia, alterations common to both osteocytes and myofibers would be an intriguing therapeutic target.

**Figure 3:**
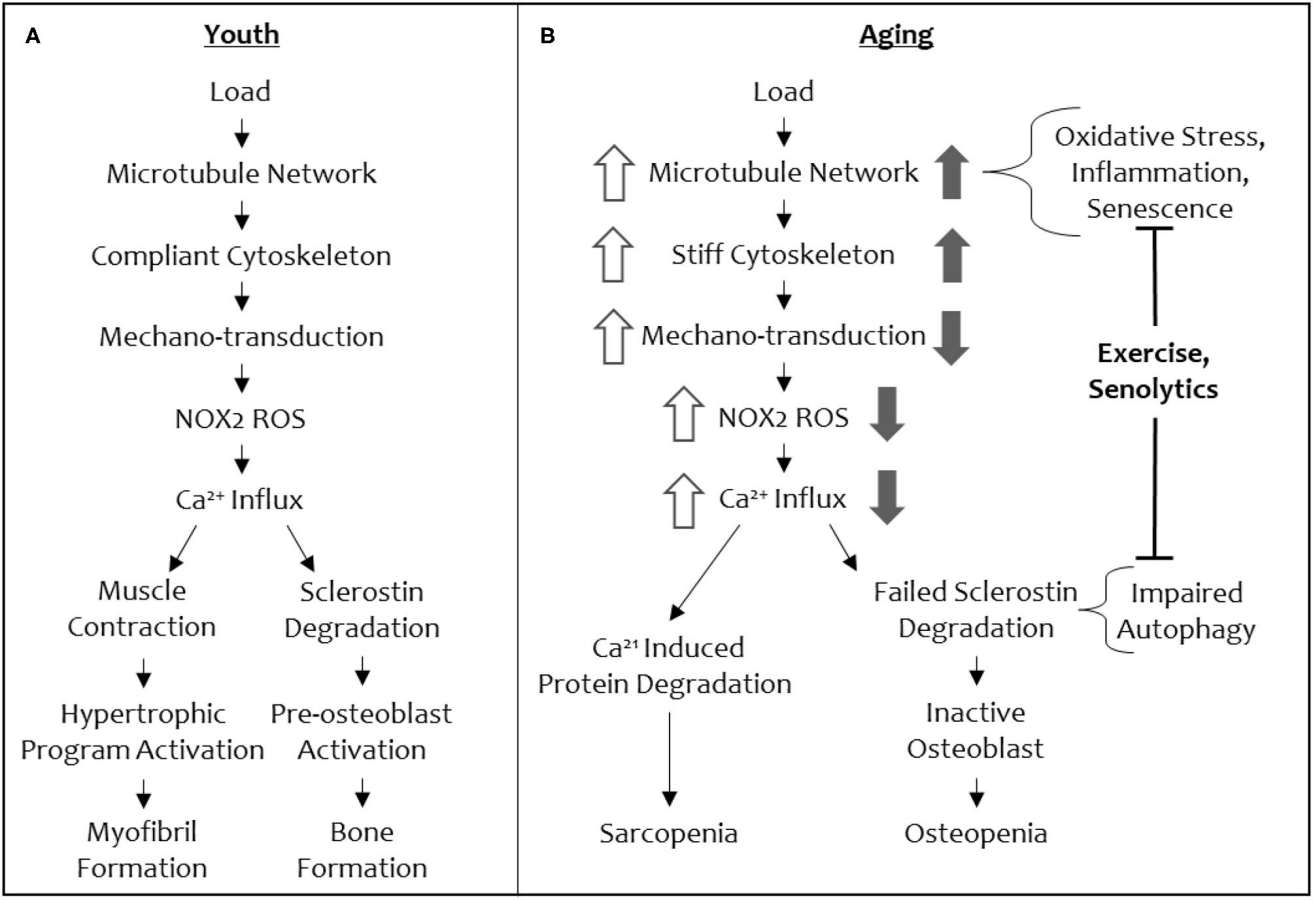
A schematic of the shared mechano-transduction pathway in youth and in aging. Depiction of the shared mechanical load pathway in youth **(A)** and in aging **(B)**. Changes in signal magnitude by aging-related phenomena are indicated in open arrows (muscle) and solid arrows (bone).

Recently, our groups have defined a mechano-transduction pathway with conserved elements in both bone and muscle (Figure 3). In both tissues, this mechano-pathway includes a subset of microtubules, which are one of the cytoskeletal proteins that regulate the biophysical properties of the cell, cytoskeletal stiffness, the production of ROS from an enzyme, NADPH oxidase 2 (NOX2), and an intracellular calcium response that governs bone and muscle function in response to mechanical loading signals (34, 35). Culmination of this mechano-transduction pathway in bone permits bone formation, increasing bone mineral density, and in muscle, this pathway ultimately converges on myofibril formation, increasing muscle mass.

Although many of these elements are shared between bone and muscle, only microtubules function in the same role in this mechano-transduction pathway in both tissues. Microtubules, along with actin and intermediate filaments, comprise the cytoskeleton and acts as a scaffold to support cells in movement and cell division among other mechanical events. Microtubule filaments, comprised of α/β-tubulin dimers, are compressive elements of a highly interconnected cytoskeleton (44). Post translational modifications to tubulin dimers can dictate particular function of a microtubule filament and its associated proteins, resulting in the notion of a “tubulin code” (45, 46). Specifically, increases in detyrosinated tubulin and acetylated tubulin allow microtubules to bend, buckle, and transmit mechanical loading signals, increase cytoskeletal stiffness, and impact muscle and bone mechano-transduction (34–36). This subset of post-translationally modified, mechano-responsive microtubules initiates a mechano-transduction cascade by translating mechanical forces into biochemical signals via their interactions with other proteins.

The conservation of microtubules as a mechano-sensory element in muscle and bone presents an intriguing opportunity to understand the concomitant loss of function in both tissues with increasing age, as well a singular therapeutic target (i.e., the microtubule cytoskeleton) to modulate for reversing osteo-sarcopenia. Central to this idea is that microtubules tune cytoskeletal stiffness and that a threshold level of stiffness is required for a mechanical load to initiate signaling (i.e., production of NOX2-ROS, calcium influx, to ultimately increase bone formation and myofibril formation and muscle hypertrophy) through this cascade (Figure 3). If the cytoskeletal stiffness is elevated beyond this threshold by specific microtubule post-translational modifications (e.g., acetylation and detyrosination) or microtubule associated proteins (e.g., Tau) then the mechano-transduction cascade is aberrantly affected (decreased mechano-transduction in bone and increased mechano-transduction in muscle). A loss of cytoskeletal stiffness below a threshold level can also impact mechano-responses in both tissues. Thus, there is a Goldilocks zone of “just right” cytoskeletal stiffness that permits musculoskeletal tissues to respond to mechanical loading cues appropriately. In normal physiology, this threshold level of cytoskeletal stiffness is set by environmental cues that adjust the stiffness to be appropriately adapted to accustomed loads, while remaining responsive to unaccustomed loads.

When cytoskeletal stiffness is increased beyond the Goldilocks zone, an opposite ROS and calcium signaling responses is initiated in muscle (excessive ROS and calcium responses) and bone (attenuated ROS and calcium responses), even though these signal transducers are shared in our mechano-pathway (Figure 3). For example, in a mouse model of Duchenne's Muscular Dystrophy, hyper-activation of NOX2 and excessive ROS (i.e., oxidative stress) is connected to muscle injury, damage, and atrophy (47, 48). Similarly, calcium influx is higher with increased cytoskeletal stiffness in muscle, disturbing calcium homeostasis, and promoting atrophy (49, 50). This makes inhibition of NOX2 and/or calcium attractive targets to alleviate the progression of sarcopenia. However, unlike muscle, bone loses mechano-activated NOX2-ROS production and TRPV4-calcium influx when cytoskeletal stiffness is pushed beyond the Goldilocks zone. Given the imperative to address osteo-sarcopenia together, rather than individually, in order to recover from the cycle of frailty, we focus the remainder of this review on the action of microtubules in aging as a potential target for a collective solution.

### Bone Mechano-Transduction

Bone resident osteocytes coordinate the actions of mechano-transduction by direct (cell-to-cell) and indirect (secreted proteins, cytokines, and signaling molecules) communication with bone building osteoblasts on the bone surface through the canalicular system, an elaborate network of “tunnels” through the bone that interconnects osteocytes and surface bone cells (51). A major effector of mechano-transduction regulate loading or disuse is the osteocyte secreted glycoprotein sclerostin.

Sclerostin is a key protein, continuously secreted by osteocytes to prevent bone formation, a measure that keeps the skeleton from becoming a metabolic sink and too heavy to move, however in response to vigorous mechanical stimulus (i.e., unaccustomed load), osteocytes reduce sclerostin abundance allowing for osteoblasts to make new bone appropriate for resisting the force experienced. In rodents, sclerostin protein, and its associated transcript, *Sost*, are decreased in response to mechanical load (52, 53) and increased in response to mechanical unloading (54, 55), making sclerostin an important gatekeeper to load-induced bone formation. While the consequences of sclerostin action in humans remains conserved, the acute relationship between sclerostin/*SOST* and mechanical load is less straight forward given loading exercises increase bone mass (56), yet circulating sclerostin protein (not bone-resident sclerostin) increases acutely after exercise (57). However, as expected based on mouse models, long term exercise reduces serum sclerostin (58). This mild discrepancy between humans and mouse responses might be partially reconciled since serum sclerostin levels, which are the outcome measure in human studies, are not necessarily temporally coupled to bone-resident sclerostin, which is typically directly measured in mouse studies (59). Additionally, unloading in young, healthy humans increases circulating sclerostin (60), an outcome that parallels mouse studies and is consistent with the role of sclerostin as a negative regulator of bone formation. A lack of mechanical loading, or disuse, can shift the balance of bone remodeling to favor osteoclast resorption and decrease bone mass.

The subset of mechano-responsive microtubules and the downstream signaling pathway that we described earlier regulates sclerostin protein abundance (35, 37). The resulting outcome of this mechano-transduction pathway is the rapid degradation of sclerostin protein by the lysosome, removing the inhibition on bone formation by osteoblasts and allowing new bone formation to occur (37).

### Muscle Mechano-Transduction

Skeletal muscle mass and performance is also regulated by mechanical demand. Brief rounds of high-load resistance exercise, outside of the accustomed homeostatic setpoint, increases the number of myofibrils and machinery involved in the contractile regulation of the muscle fiber. Recruitment of these organelles/proteins increase the muscle fiber cross sectional area, resulting in muscle hypertrophy (morphology) and increased force production (function) (61–63). Central to these hypertrophic changes are mechanical loading cues (contraction or stretch) that elicit mechano-transduction signaling effectors (IG-F1, Ang-II, ROS, calcium, and phosphorylation) to regulate pathways in the muscle fiber [calcineurin-NFAT, rapamycin-sensitive mTORC1, and mitogen-activated protein kinase (MAPK)] increasing capacity for transcription and translation (64, 65). Additionally, the fusion of myogenic stem cells (satellite cells) to existing myofibrils contributes myonuclei necessary to accommodate the increased transcriptional needs of the hypertrophic program, and assist in repair of the muscle fiber if injury occurred (66). On the other hand, unaccustomed low-load exercise generates improved mitochondrial function and overall oxidative capacity, with little change in muscle fiber size, myonuclei number, or muscle mass, highlighting the mechanical sensitivity, and specificity of the hypertrophic response (67–69).

Similar to the bone, muscle also adjusts its mass to decreased load conditions of bedrest, immobilization, and inactivity. In muscle, the reduction in accustomed load promotes a decline in mass (atrophy) and function by reducing the very same transcription and translation required for hypertrophy. The reduction in myofibrillar content and supporting proteins yields smaller myofibers and thus muscle mass to match the reduced mechanical demand (70–72). Central to age-related atrophy are increases in pro-inflammatory cytokines, excess cytosolic calcium, and oxidative stress that impairs the hypertrophic responses and underscores a decrease force production independent of the loss in muscle mass (73–77). Here we propose these signals also perpetuate the aging related deficits in mechano-transduction.

## Musculoskeletal Aging: Problems With Age Related Cellular Stress

Numerous interdependent aging related factors, including senescent cells, chronic low-grade inflammation, oxidative stress, and changes in autophagy may all influence bone and muscle. We will first describe their broad impacts on tissue function before discussing their influence on the cytoskeleton and potential impacts on bone and muscle mechano-transduction.

A major driver of these aging related changes is the accumulation of senescent cells, which activate the innate immune system, promote inflammation, and oxidative stress. Cellular senescence is a protective feature of healthy aging, where cells that have exhausted their abilities to function call attention to themselves in order to be cleared from their environment. A senescent cell acquires the senescence associated secretory phenotype (SASP) which includes secreting numerous inflammatory cytokines and ROS among other traits, to invite innate immune cells to safely destroy the senescent cell (78, 79). During aging, the body becomes inundated with chronically senescent cells such that SASP overwhelms local tissues creating a stressful environment that can change the function of remaining cells. Indeed, the aging dependent increase in senescent cells, the SASP factors secreted by these cells, and the activation of the innate immune system contribute to oxidative stress and pro-inflammatory environment of aging. Consequently, senescence has been attributed to depletion of osteoprogenitors (80), whereas clearance of senescent cells in bone prevents age-related bone loss (81). Acquisition of a senescent phenotype also prevents muscle repair in response to injury, and promotes muscle wasting (82, 83).

A consequence of the accumulation of senescent cells is the change from physiologic ROS signaling to pathologic oxidative stress. Cells use reactive oxygen species (ROS) and reactive nitric species (RNS) to trigger biological events, including mechano-transduction. However, the production of these highly reactive free radicals is rapidly buffered or detoxified by a cellular counter-response known as reduction-oxidation (redox) buffering. When ROS and RNS signals exceed this buffering capacity it becomes damaging oxidative stress. Part of cellular function is adaptation to stress, which can encompass the imbalance between oxidative signaling and oxidative buffering that leads to cellular dysfunction. Aged cells are frequently under oxidative stress because changes in gene expression, impaired mitochondrial function (84), and excessive exposure to oxidative molecules, which occurs during aging-related cellular senescence (85), overwhelm the redox buffering capacity of the cell. Ultimately, this results in oxidative damage to macromolecules disrupting cell cycle progression, autophagy, and gene transcription while promoting inflammation and apoptosis (86). The presence of oxidative stress in aging has been linked to poor bone mineral density measures in men and women, with loss of sex hormones as a compounding factor (87–89). Oxidative stress is a keystone feature of osteo-sarcopenia (71, 77), and correlates to reduced gait speed and frailty (90).

In aged individuals, chronic, low-grade inflammation due to overexpression of pro-inflammatory cytokines such as IL-1, IL-6, and TNF, Baylis et al. (91) has been referred to as inflammaging, and contributes to a wide range of diseases, including cancer, arthritis, vascular disorders, and musculoskeletal dysfunction (92, 93). For example, cross-talk between inflammatory cytokine signaling and pro-osteoclast activating receptor activator of nuclear factor kappa-B ligand (RANKL) stimulates excessive bone resorption (94), generating a net loss of bone mineral density. Chronic inflammation is also associated with osteopenia in bone (95) and sarcopenia in muscle (96), and in addition is highly correlated with frailty, disability, and mortality (97). Inhibition of IL-6 (SASP component) prevents muscle atrophy (98).

Additionally, aging-related inflammation can trigger skeletal progenitor cell dysfunction, a phenotype that was uncoupled to chronological age using a murine genetic knockout of *NFKb1*, a transcription factor that is prominent in initializing pro-inflammatory cascades, and restored in aged mice using pharmacological intervention (99). Targeting inflammaging with metformin improved age-related deficits in autophagy and redox buffering (100). Metformin has also been shown to regulate bone marrow mesenchymal stem cells and osteogenic differentiation to improve trabecular bone formation around dental implants in osteoporotic rats (101). The effect of metformin on bone mass is provocative; while there are exceptions (102), in most studies it appears to be that metformin improves bone microstructure in rodent ovariectomized osteoporosis (103–105) and stimulates osteoblast mineralization *in vitro* through AMP-activated protein kinase (AMPK) (106). While metformin has shown some interesting benefits as a target of aging in mice, significant caveats remain, as the benefit of metformin blunts muscle hypertrophy in response to resistance training in aging humans (107). The benefits in mice do not directly translate into benefits for humans. Regardless, metformin's action in rodents is consistent with an important role for inflammation and oxidative stress, and defects in autophagy in aging related deficits in musculoskeletal tissues.

Autophagy is a lysosome-dependent mechanism through which cells regulate cytoplasmic turnover, and an important component of cellular proteostasis. Proteostasis, the balance between protein production and degradation, is held in check in part through the ability of the autophagosome (in addition to the proteasome and endoplasmic-reticulum-associated protein degradation) to remove or recycle malfunctioning, damaged, or unneeded proteins and organelles via fusion with lysosomes (108). In aging, autophagy is reduced, due in part to decreased acidity of the lysosome (109) and decreased expression of autophagy-related proteins (110). This may lead to protein aggregation, which is commonly associated with a variety of disorders and diseases (111). Furthermore, genetic deletion of proteins associated with autophagy can produce an “aged” phenotype (112), typically characterized by a decrease in bone mass (113, 114), or decreased muscle strength (115, 116), suggesting the incontrovertible role that autophagy plays in aging.

Notably, autophagy is a downstream consequence of mechano-transduction in both muscle and bone. Mechanical load stimulates autophagy in bone and controls the load-dependent degradation of sclerostin (37). Defects in lysosome activity can impact bone health (117) at least in part through the failure to remove the inhibitor of bone formation, sclerostin (37). In muscle, mechanical load also converges on autophagy with mechano-transduction regulated mTORC1 translocation to the lysosome necessary to elicit the hypertrophic signaling cascade (118). In fact, NOX2-dependent ROS signaling activates lysosomes and autophagic flux, but excess ROS can inhibit autophagy and impact muscle mass and function (48). Through this axis, alterations in autophagy due to aging could contribute to muscle deficits through diminished hypertrophic program activation (119–121).

## Influence of Aging Related Changes on the Cytoskeleton and Musculoskeletal mechano-transduction

Interestingly, many of the factors associated with aging seem to also influence the microtubule-based mechano-sensor that is conserved in the musculoskeletal system (Figure 1). The cumulative impact of many of these aging related changes, which reportedly increase cytoskeletal stiffness microtubule post translational modification in non-musculoskeletal cells, may partially explain the altered mechano-responsiveness of aged muscle and bone. Many of these aging related factors may push microtubules and cytoskeletal stiffness outside of the mechano-responsive Goldilocks zone and into a pathological gain (muscle) or loss of function (bone) response that deteriorates tissue quality. This suggests microtubules may be a common therapeutic target to improve muscle and bone function in aging. However, it is important to acknowledge that many mechano-transduction pathways are operant in these tissues (53, 122–128) and the sum total of effects of aging related factors on tissue homeostasis certainly extend beyond impacts on microtubule mechano-transduction. Rather, we wish to speculate on microtubules and mechano-transduction as provocative additional players in the onset of osteo-sarcopenia and examine how several aging related factors influence this subset of mechano-sensitive microtubules to lead to an alteration of bone and muscle mechano-transduction.

Increased cytoskeletal stiffness appears to both regulate and be a consequence of cellular senescence (129). Using progeroid syndromes as models to study pre-mature senescence, evidence supports that multiple mechano-transduction pathways become dysregulated with the onset of this aging phenotype (130). Reorganization of the microtubule network is a specific cytoskeletal consequence of senescence, including increased microtubule stability and increased tubulin acetylation (131). Indeed, targeting senescent cells with senolytics has proven to be an incredibly effective generalized therapy to improve musculoskeletal health in rodent models (98, 132, 133). We predict that removal of senescent cells through senolytics is likely to improve the ability to activate mechano-transduction in musculoskeletal tissues.

Likewise, oxidative stress can impact the cytoskeleton, including microtubules (134). Chronic oxidative stress encourages more frequent microtubule repair, generating a dense microtubule network in cardiomyocytes over time (135). Similarly, pro-inflammatory cytokine signaling promotes increased cytoskeletal stiffness (136) and microtubule rearrangement (137). This potentially interferes with microtubule sensation of load (insensitive in bone, hypersensitive in muscle) and initiation of ROS signaling (reduced in bone, enhanced in muscle) for the mechano-transduction pathway. Indeed, inflammation inhibits mechanically-induced calcium production in osteocytes (138), while resolution of inflammation restarts the anabolic Wnt/β-catenin signaling pathway and activates osteoblasts (139).

The drug colchicine presents an appealing opportunity to both ameliorate inflammation and rescue microtubule driven cytoskeletal stiffness. Functional consequences of colchicine action include inhibition of neutrophil migration and inflammatory signal transducers, while its direct action prevents polymerization of microtubules (140). In fact, colchicine shows promise as an effective measure against cardiovascular events, given a feature of the disease is an increase in microtubule density and detyrosination in cardiomyocytes (141, 142). Further support for targeting microtubules as a potential therapeutic for mechano-transduction in aging can be found with parthenolide. A specific inhibitor of the enzyme that generates detyrosinated tubulin and therefore also cytoskeletal stiffness, exposure to parthenolide rescued cytoskeletal stiffness and mechano-transduction in myocytes and osteocytes *in vitro*, and prevented eccentric contraction-induced injury in a murine model of Duchenne's muscular dystrophy (34, 35). Either of these pharmacologic interventions offer the opportunity to transform a stiff cytoskeletal network back into the mechano-responsive Goldilocks zone, as a way to improve musculoskeletal mechano-transduction.

It is worth noting that the effects of oxidative stress on microtubules may not be perfectly straightforward however. In contrast to chronic oxidative stress, acute treatment of neurons with glutathione disulfide, a pro-oxidant, causes neurons to retract cell processes and reduce the amount of tyrosinated α-tubulin relative to vehicle treated cells (143). This parallels aging-related changes observed in osteocytes in bone. Osteocytes, much like neurons, have an extensive cell process network that resides in the lacunar-canalicular network throughout bone (144). Additionally, these long osteocyte cell processes are mechano-responsive elements (145). Although the effect of oxidative stress on osteocyte microtubules has not been investigated directly, if osteocytes were to behave like neurons (and yes, they do share a number of physiological similarities (146) then an acute bolus of ROS might deplete mechano-sensing structures resulting in diminished mechano-transduction. Consistent with this predicted effect, osteocytes in aged bone have fewer neuron-like cell processes, and form fewer intercellular connections, which would be predicted to impair their ability to coordinate new bone formation (147) In this condition of advanced aging, strategies to improve redox buffering capacity could act to prevent this catastrophic microtubule loss and maintain the cytoskeleton in the mechanically responsive Goldilocks zone. Alternatively, clinically approved microtubule targeted therapeutics that increase microtubule density and promote its modification by detyrosination and acetylation (i.e., paclitaxel) may effectively protect the microtubule network as recently demonstrated in experimental ischemia reperfusion injury (148, 149), and in models of neurodegeneration (29).

The implication of lysosome activity and autophagy as they relate to microtubules are abundant. With respect to our mechano-signaling pathway, lysosome activity and autophagy are influenced by mechanical loading in both bone and muscle and therefore are affected in these cells based on loading stimuli. Additionally, lysosomes use microtubules to position themselves in the cell to carry out their function. Thus, aging related changes in microtubules, influenced by cellular senescence, oxidative stress, and inflammaging, likely affect autophagy independent of mechano-signaling as well, because microtubules themselves affect lysosome trafficking (150). Lysosomes are positioned throughout the cell by microtubule associated motor proteins, kinesins and dynein (151, 152). Specific microtubule post-translational modifications (acetylation, detyrosination, and polyglutamylation) confer preference for particular motors (153–155) while certain MAPs (Tau and MAP2) impede motor movement (156, 157). Given the relationships between microtubule post-translational modifications and motors, and microtubule motors and lysosome movement, it is not surprising that post-translational modifications or MAPs could spatially restrict lysosomes to a region of the cell, such as detyrosination (158). This sequestration potentially limits lysosome activity and autophagy. Since degradation of sclerostin protein by the lysosome is an outcome of mechano-transduction in bone that leads to bone formation, and mTORC1 association with the lysosome is an outcome of mechano-transduction in muscle that leads to hypertrophy, we speculate that improvements to bone and muscle catabolism can be achieved through targeting aging-dependent influences on microtubules, synergistically activating both mechano-transduction and alleviating impaired autophagy.

## Exercise

Exercise is among the most impactful interventions for improving overall health. In fact, the beneficial effects of exercise on longevity, overall health, and musculoskeletal health in the elderly are well-documented (159–163). Evidence that musculoskeletal function (i.e., grip strength, gait speed, fracture) appears a sentinel indicator of age-related frailty has drawn attention to the mechanisms by which musculoskeletal tissues are altered in aging.

Aging is acknowledged as a systemic process that impacts all tissues albeit with a varied trajectory of severity. Common to cellular aging in each tissue is increased cellular senescence, oxidative stress, inflammation, and impaired autophagy. While exercise exerts numerous systemic effects, the cellular benefits are underscored by reduced cellular senescence, oxidative stress, inflammation, and improved autophagy. Here we focus on how these cellular benefits within osteocytes and muscle fibers may act though improving age-altered mechano-transduction to elicit their benefits in bone and muscle.

Exercise is proficient at the clearance of senescent cells. Exercise is connected to reductions in senescence associated markers, such as p16 and p21 among others, in human muscle (164). Others have postulated that exercise potentially reverses osteocyte senescence and promotes osteocyte viability even though it did not improve bone mass (165). As senescent cells are a significant source of oxidative stress and inflammation in bone and muscle that drives increased cytoskeletal stiffness in their cells, clearance of senescent cells by exercise will undoubtedly improve bone and muscle health. We reason that a portion of this improvement will be through a partial normalization of microtubule mechano-transduction.

The benefit of exercise is also from the oxidative stress and inflammation generated as a consequence of exercise itself. It is paradoxical that ROS and inflammatory signals act as intermittent low-grade stressors to elicit the transcriptional regulation of redox buffering and anti-inflammatory cytokines (68) to protect the system long-term for benefit (161, 166). These benefits of acute stress are also underscored by evidence that targeting inflammation or oxidative stress alone can be deleterious to exercise adaptation in young recreational athletes. Further evidence is in the elderly treated with metformin, and inhibitor of oxidative stress and inflammation, and exercise, show smaller gains to those exposed to only exercise (167).

Additional benefits of exercise include the stimulation of autophagy and mitochondria protein synthesis through the PCG-1α pathway in aged rodent muscle models (168, 169), and increased mitochondrial capacity in elderly humans (170). In both murine and human muscle, lifelong exercise in aged cohorts produced protein expression profiles of increased autophagy, including microtubule associated proteins LC3II/LC3I and sequesterome-1 (p62) (171). In muscle, lysosomal activation by exercise goes through a number of pathways including the AMPK/ULK1 (Unc-51-like autophagy activating kinase 1) axis, and the AKT/FOXO3 axis (172–174). Intriguing is the evidence in the failing heart where AMPK activation by 5-aminoimidazole carboxamide ribonucleotide (AICAR) or expression of constitutively active AMP (CA-AMPK) reversed the pathologic remodeling of the microtubule network (i.e., reduced microtubule density and level of detyrosinated tubulin) (175). Given that these interventions model the effects of vigorous exercise training, testing their impact in the musculoskeletal system toward improving mechano-transduction, is of high interest.

In total, the benefits of exercise in the elderly are many. We speculate that among the benefits caused by exercise may be, at minimum, a partial reset of the microtubule cytoskeleton to a mechano-responsive range. While exercise is a clinically significant intervention with regard to musculoskeletal frailty, it is not a panacea. Even with exercise-induced reductions in senescence-associated ROS and inflammation, these consequences of aging are not completely eliminated. We predict that while the systemic benefits of exercise to remove senescence, decrease oxidative stress and inflammation and promote autophagy, exercise alone in unlikely to be sufficient to yield complete restoration of bone and muscle mechano-transduction.

However, not all exercise is equal. The physiological benefits of resistance exercise may be different than low intensity exercise. While low intensity exercise may reap some systemic effects on inflammaging, resistance exercise (unaccustomed load) is necessary to maintain or even improve bone and muscle mass. Resistance exercise, in contrast to low intensity or cardiovascular type exercise, are efficacious in both the young and the elderly for increasing bone and muscle mass (176–181). But for an aging population, osteo-sarcopenia serves as a barrier to executing the magnitude of unaccustomed load necessary for commencement of anabolic programming. Therefore, the key is to try to keep activity as high as tolerable to ensure that the lack of mechanical cues does not lead to unaccustomed unloading which initiates bone and muscle atrophy. While walking and other low impact exercise are not typically considered sufficient load to induce bone and muscle formation during youth, in aging, it could be advantageous to stave off further tissue loss by shifting activity levels to an acceptable accustomed load which preserves tissue maintenance (lazy zone) and away from disuse. Optimal musculoskeletal health during aging is most likely to occur when exercise benefits are overlaid with unaccustomed loading, which may synergistically benefit muscle and bone quality. However, as in youth, the homeostatic set point will adjust to static exercise, and progressive resistance training is the most useful intervention to promote muscle and bone anabolism.

## Discussion

In summary, we present the concept that among the deficits contributing to musculoskeletal frailty is a defect in mechano-transduction. Mechano-sensors present in bone and muscle cells are likely impacted by systemic changes in aging, like senescence, low grade chronic inflammation, oxidative stress, and defects in autophagy. We speculate that these aging related changes tip the homeostatic setpoint for an unaccustomed load to initiate anabolic effects in muscle and bone to be increased. We support that this altered mechano-setpoint accelerates the cycle of frailty. Deficits in mechano-transduction propagate the cycle of frailty through failure to stimulate anabolic programs and through progression of catabolic pathways which lead to osteo-sarcopenia. Aging-related declines in mechano-transduction are exacerbated by reductions in activity levels. While exercise may help to combat detrimental elements of aging in bone and muscle, it may not suffice to restore bone and muscle formation alone, and unaccustomed loading through progressive resistance training is most certainly needed to improve muscle and bone mass/quality.

Importantly, understanding the molecular mediators of the aging related alteration of mechano-set point provides key therapeutic targets to preserve or increase bone and muscle mass in aging. Truly, reducing senescence, inflammation, oxidative stress, and defective autophagy through exercise or pharmacology, combined with targeting elements of common mechano-transduction cascades to improve mechano-transduction gives us our best strategy to improve musculoskeletal frailty, disrupt the cycle of frailty, and restore or preserve musculoskeletal health in the context of aging. To return to our earlier analogy, we posit that synergistic targeting of aging related systemic changes and mechano-transduction thresholds, such as by targeting microtubules, will allow aged individuals to achieve the same range of anabolic response gained from unaccustomed mechanical loading, such as weights on a squat bar, without having to compensate by providing unachievable loads.

While the aim of this review is to highlight the global changes that occur during aging which impact mechano-transduction in bone and muscle particularly at the shared microtubule level, we want to acknowledge that they are but a portion of the mechano-sensing apparatus. Each of these mechano-sensors or mechano-transduction pathways, while not described in any depth here, are all reasonable targets for affecting musculoskeletal frailty. Furthermore, even within the microtubule-dependent mechano-sensing pathways in muscle and bone, there is much yet to be learned about the role of each tubulin post-translational modification and the prevalence of these modifications in aging. Additionally, the other shared elements of our mechano-transduction pathway, NOX2-ROS and calcium influx, also contain potential for mediating osteo-sarcopenia. Indeed, many factors, like myokines, signal between bone and muscle, and are additional factors to consider when discussing reversal of the cycle of frailty (182–185). Regardless, much future investigation is needed to understand, test and develop targets shared by muscle and bone or communicated between muscle in bone to combat aging.

## Author Contributions

All authors contributed to the conceptualization and writing of this review article.

## Funding

This work was supported funding from NIH (AR071614, JS and CW; AR063631, JS; AR071618 CW).

## Conflict of Interest

JS and CW holds two patents related to this work. One for the custom fluid shear device used for these experiments US Patent No US 2017/0276666 A1 and a second for the targeting microtubules part of this mechano-transduction pathway to improve bone mass US Patent No US 2019/0351055 A1. CW also has another patent pending on colchicine analogs to treat musculoskeletal disorders PCT/US2018/038300. The remaining authors declare that the research was conducted in the absence of any commercial or financial relationships that could be construed as a potential conflict of interest.

## Publisher's Note

All claims expressed in this article are solely those of the authors and do not necessarily represent those of their affiliated organizations, or those of the publisher, the editors and the reviewers. Any product that may be evaluated in this article, or claim that may be made by its manufacturer, is not guaranteed or endorsed by the publisher.
